# How addicted are newly admitted undergraduate medical students to smartphones?: a cross-sectional study from Chitwan medical college, Nepal

**DOI:** 10.1186/s12888-020-02507-1

**Published:** 2020-03-02

**Authors:** Sirisa Karki, Jaya Prasad Singh, Gita Paudel, Sushma Khatiwada, Sameer Timilsina

**Affiliations:** 1 Department of Pharmacology, Tribhuvan University, Chitwan Medical College, Post Box No: 42, Bharatpur-5, Chitwan Nepal; 2 School of Public Health, Chitwan Medical College, Bharatpur-5, Chitwan Nepal; 3 Department of Anatomy, Tribhuvan University, Chitwan Medical College, Bharatpur-5, Chitwan Nepal; 4 Department of Physiology, Tribhuvan University, Chitwan Medical College, Bharatpur-5, Chitwan Nepal

**Keywords:** Smartphone, Addiction, Undergraduate, Medical

## Abstract

**Background:**

Increasing smartphone use among adolescents in todays’ world has made this handy device an indispensable electronic tool, however, it comes at a price of problematic overuse or addiction. We aim to investigate the prevalence of smartphone addiction among undergraduate medical students and explore its association with various demographic and personal factors.

**Methods:**

A pool of 250 undergraduate students completed a survey composed of socio-demographics information, smartphone-use related variables and 10-point Smartphone Addiction Scale-Short Version in February 2019.

**Results:**

Smartphone addiction among medical students was estimated at around 36.8% with higher percentage of male smartphone addicts. Phubbing was reported by 37.6% participants with more than 60% reporting overuse. Statistically significant association was observed between smartphone addiction and gender and overuse. Self-acknowledgement of addiction was found to be the biggest predictor of smartphone addiction.

**Conclusion:**

This study provides preliminary insights into smartphone use, smartphone addiction and various factors predicting smartphone addiction among early undergraduate medical students from Nepal, which should be extended in future studies. Education policymakers and educators need to develop some strategies encouraging student’s smartphone utilization to enhance academic performance.

## Background

With the advancement in science and technology, the way we live, our life style and everything including the way we remain connected to people, has changed drastically. The advances in telecommunication means has evolved our lives from simple to becoming simply complicated. Technology has enabled people to access the previously thought inaccessible information at ones’ fingertips.

As of recent times, people living in the lesser developed world have encompassed a massive transformation in mobile and internet technology. Smartphone has reached a substantial 2.1 billion people worldwide [1]. Studies have shown that young children as less as 2 years to 17 years use smartphone more than the older generation, although its ownership increases with age [2]. In Nepal, Nepal Telecom (Nepal Doorsanchar Company Limited) is the incumbent operator, owned by the government of Nepal that provides the telecommunication services. Internet service started with the beginning of the century and in less than 17 years, its penetration has reached over 58% and rising [3, 4].

Increasing smartphone use among adolescents in todays’ world has made this handy device an indispensable electronic tool. Smartphone applications can be applied in education, [5] health [6], smoking cessation [7], alcohol usage monitoring [7] and virtual reality therapy [8]. However, it comes at a price of problematic overuse or addiction. Individuals with prolonged and excessive smartphone use present with symptoms like self-absorption, inability to control craving, disturbances in daily schedule ignoring adverse consequences and withdrawal. These presenting symptoms are similar to those observed in patients with “Substance-Related and Addictive Disorders” as described in Diagnostic and Statistical Manual of Mental Disorders (DSM-5) [9]. Like pathological gambling, which actually has been stated in DSM-5 as behavioral addiction, internet gaming disorder and smartphone addiction are on line awaiting sufficient evidence [9]. Several studies conducted across the world show addiction of children, adolescents and university students to the smartphone. Students use smartphone as a tool for studying, communicating with friends and family and study purpose. Easy internet connectivity along with user-friendly advanced technology help students easily access required study materials. On the other hand, despite of its apparent benefits, excessive smartphone and internet use can lead to anxiety, [10] attention deficit [11], sleep disturbance, [12] poor quality of life, [13] and poor academic growth. Smartphone addiction has been investigated among medical students reporting hindrance to academic activities and overall growth and performance. In the present study, we aim to evaluate the status of smartphone addiction among medical undergraduate students of Chitwan Medical College and study its association with various demographic variables.

## Methods

A total of 250 first year undergraduate students at Chitwan Medical College, Bharatpur, Nepal were recruited from the pool of “School of Medicine”, “School of Dental Surgery”, “School of Nursing” and “School of Allied Sciences”. All 261 first year undergraduate students enrolled at the college were invited to participate in the study in February 2019, but only 95.7% (250) of students entered the survey. Prior to the survey, researcher briefed the students about the objectives and procedure of the study. The participants were also informed about the voluntary nature of participation and non-participation would not bear any academic consequences. A written consent was obtained from the students for participation in the study. Ethical clearance for the study was obtained from Institutional Review Committee of Chitwan Medical College (ref no: CMC-IRC/075/076–123). The first section of the survey included demographic information (age, gender, faculty, duration of smartphone-use per day during weekdays, purpose of smartphone use, and self-evaluation of smartphone addiction) and the second section included a 10 item shorter version of smartphone addiction scale (SAS-SV). Participants were instructed to rate the items on a 6-point Likert scale, 1 = “strongly disagree”, 2 = “disagree”, 3 = “slightly disagree”, 4 = “slightly agree”, 5 = “agree” and 6 = “strongly agree” so that the addiction score ranged from 10 to 60. As recommended by the guideline, an overall score of greater than 33 in males and greater than 31 in females was considered addiction [14]. The SAS-SV considers 5 parameters of smartphone addiction; 1. Disturbance in daily life (3 questions), 2. Withdrawal (4 questions), 3. Overuse (1question), 4. Tolerance (1 question) and 5. Virtual relationship (1 question). Using smartphone for longer than 5 h daily during weekdays was labeled as “overuse”. The purpose of smartphone use included 3 multiple choice options namely communication, social networking and gaming and study purposes. Participants were also instructed to self-evaluate their addiction status. Statistical analysis was done using SPSS-20. Numerical values were expressed as mean ± SD and categorical variable as percentage. Chi-square test was used to seek association between demographic variables in addicted and non-addicted samples. Logistic regression analysis was performed for multivariate analysis to seek the association between smartphone-use variables and smartphone -addiction.

## Results

The study included 250 undergraduate students: 38.8% males (97/250) and 61.2% females (153/250) with mean age of 19.7 ± 1.68 years (range 18-29 years). The pool of participants included 87 students from school of medicine, 51 from school of dental surgery, 96 from school of nursing and 16 from school of allied sciences.

Most of the participants (224) attended private or boarding schools before joining medical school. 74% (185) of the participants currently resided in hostel. Smartphone was commonly found to be used for communication, social networking, gaming and study purposes (26.8%). 26.8% (67/250) of the participants self-rated themselves as addicted to smartphone, 42% (105/250) did not rate themselves as addicted and the rest 31.2% (78/250) had no opinion of the same (Table 1).

**Table 1:** Characteristics of study participants

Variables	Frequency (%)
Gender	
Male	97 (38.8%)
Female	153 (61.2%)
Faculty	
School of medicine	87 (34.8%)
School of Dental Surgery	51 (20.4%)
School of Nursing	96 (38.4%)
School of Allied sciences	16 (6.4%)
Past Educational Institute	
Private/ Boarding School	224 (89.6%)
Government/ Public School	26 (10.4%)
Place of residence	
Hostel	185(74%)
Day-scholar	65 (26%)
Reasons for using Smartphone	
Communication	16 (6.4%)
Social networking and gaming	23 (9.2%)
Studying	12 (4.8%)
Communication, social networking and gaming	25 (10%)
Social networking, gaming and study purposes	59 (23.6%)
Communication and study	48 (19.2%)
Communication, social networking, gaming and study purposes	67 (26.8%)
Self-Perception of Smartphone addiction	
Yes	67 (26.8%)
Don’t Know	78 (31.2%)
No	105 (42%)
Duration of Smartphone use (weekdays)	
> 5 h/weekdays	42 (16.8%)
≤5 h/weekdays	208 (83.2%)

Indications of disturbance in daily life was reported by over 60% participants. The study also reported nomophobia among 72.4% of participants. Likewise, phubbing was reported among 37.6% participants. Overuse was reported by 60.8% participants. Tolerance was observed in 42.8% participants who accepted peoples concern about excessive smartphone use (Table 2).

**Table 2:** Prevalence of smartphone addiction (SAS-SV) symptoms among study participants

Symptoms	Items	n (%)
Disturbance in daily life	I have missed planned work due to Smartphone use.	108 (43.2)
	I have a hard time concentrating in class, while doing my assignments or while working due to Smartphone use.	64 (25.6)
	I feel pain in the wrist or on the back of my neck due to smart phone use.	98 (39.2)
Withdrawal	I will not be able to stand not having a Smartphone.	142 (56.8)
	I feel impatient and fretful when I am don’t have my Smartphone with me.	109 (43.6)
	I have Smartphone on my mind even when I am not using it.	73 (29.2)
	I will not give up using my Smartphone even when my daily life is already greatly affected by it.	36 (14.4)
Virtual relationship	I constantly check my Smartphone so as not to miss conversation between other people on twitter, Facebook, Viber, WeChat, snapchat.	94 (37.6)
Overuse	I feel like I am using my Smartphone more than I had intended.	152 (60.8)
Tolerance	The people around me tell me that I use my Smartphone too much.	107 (42.8)

Smartphone addiction was found among 36.8% (92/250) of the participants with equal numbers of male and females (46). A higher percentage of males were found to be addicted to smartphones (M = 47.42% F = 30.06%). The average addiction score among males was 30.23 ± 9.40 and that among females was 28.89 ± 8.63. A higher average addiction score was obtained among females.

Participants using smartphone for communication, study, gaming and social networking had the higher addiction scores (40.37 ± 6.04). Participants accepting self-addiction reported highest addiction scores followed by those who used smartphones for longer duration during weekdays (Table 3).

**Table 3:** Smartphone addiction (total SAS-SV) and participants’ characteristics

Parameters	Overall SAS-SV score mean ± SD (n)	Addiction Score mean ± SD (n)
Gender		
Male	30.23 ± 9.40 (97)	38.21 ± 5.63 (46)
Female	28.89 ± 8.63 (153)	39.36 ± 5.45 (46)
Faculty		
School of Medicine	30.29 ± 9.23 (87)	38.49 ± 5.50 (39)
School of Dental Surgery	28.66 ± 10.85 (51)	40.69 ± 7.10 (17)
School of Nursing	28.34 ± 7.35 (96)	38.10 ± 3.47 (28)
School of Allied Sciences	30.62 ± 10.09 (16)	38.86 ± 4.54 (8)
Past Educational Institute		
Private/ Boarding School	29.67 ± 9.17 (224)	39.06 ± 5.58 (85)
Government/ Public School	27.15 ± 6.30 (26)	35.42 ± 3.65 (7)
Reasons for using Smartphone		
Communication	24.43 ± 5.09 (14)	33.11 ± 0.63 (2)
Social networking and gaming	28.34 ± 8.43 (23)	38.41 ± 3.92 (7)
Studying	28.91 ± 6.30 (12)	34 ± 2.99 (5)
Communication, social networking and gaming	26.44 ± 9.12 (25)	36.50 ± 5.07 (8)
Social networking, gaming and study purposes	28.77 ± 9.49 (59)	38.98 ± 6.54 (20)
Communication and study	29.64 ± 7.85 (48)	37.77 ± 3.88 (18)
Communication, social networking, gaming and study purposes	32.56 ± 9.59 (67)	40.37 ± 6.04 (34)
Duration of Smartphone use / weekday		
> 5 h	32.66 ± 10.04 (42)	39.72 ± 6.95 (23)
< =5 h	28.75 ± 8.58 (208)	38.46 ± 5.01 (69)
Self-Perception of Smartphone addiction		
Yes	35.55 ± 8.18 (67)	39.80 ± 6.66 (44)
No	25.17 ± 6.87 (105)	36.86 ± 2.74 (15)
Don’t Know	29.85 ± 8.94 (78)	38.26 ± 4.45 (33)
Residence at present time		
Hostellers	28.81 ± 8.80 (185)	38.89 ± 5.18 (32)
Day-scholars	31.92 ± 9.20 (65)	38.72 ± 5.79 (60)

Among 92 smartphone addicted participants, 44 (65.7%) had self-rated themselves positively for addiction. 15(14.3%) participants who did not self-accept were found to be addicted, and 33 (42.3%) of those who did not opine were addicted to smartphone. Smartphone addiction was found to be associated with gender, duration of use and self-acceptance of smartphone addiction. It was not associated with faculty, past educational institute and place of residence at current time (Table 4).

**Table 4:** Association between smartphone addiction and participants’ characteristics

Variable	Addiction		_*χ*_2	*p*-value
	Addicted n	Not-addicted n		
Gender			7.690	0.006
Male	46	51		
Female	46	107		
Faculty			6.278	0.099
School of Medicine	39	48		
School of Dental Surgery	17	34		
School of Nursing	28	68		
School of Allied Sciences	8	8		
Past Educational Institute			1.217	0.270
Private/ Boarding School	85	139		
Government/ Public School	7	19		
Duration of Smartphone use / weekday			7.003	0.008
> 5 h	23	19		
< =5 h	69	139		
Self-Perception of Smartphone addiction			47.915	< 0.001
Yes	44	23		
No	15	90		
Don’t Know	33	45		
Residence at present time			41.145	0.420
Hostellers	32	153		
Day-scholars	60	5		

Male undergraduate medical students were more likely to be addicted than females (OR: 1.99). Self-acceptance of addiction was the biggest predictor of smartphone addiction among the studied variable (OR: 11.088) (Table 5).

**Table 5:** Multiple Logistic Regression between smartphone-use variables and addiction

Variable	B	*p*-value	OR	95% CI
Gender				
Male	0.688	0.027	1.990	1.082–3.65
Female			1	
Duration of Smartphone use / weekday				
> 5 h	0.157	0.690	1.17	0.541–2.531
< =5 h			1	
Self-Perception of Smartphone addiction				
Yes	2.406	< 0.001	11.088	5.715–24.039
Don’t know	1.331		3.786	1.835–7.814
No			1	

## Discussion

The smartphone technology is sweeping the world at an alarming rate but the advancement comes at a price; the risk of jeopardizing social life and being addicted to the virtual world. The mobile phone penetration in Nepal is 123% with mobile internet penetration around 58% [2]. The fondness of owning, overusing and engaging in virtual social life has massively engulfed the youth of the twenty-first century. In the present study, the smartphone was owned by all 250 (100%) participants. Smartphone addiction was found to be around 36.8% similar to a study among adolescents in India [9] but slightly higher than prevalence of internet addiction (30%) among medical students worldwide [15]. The similarity could have been because of similar socio-economic conditions. Nomophobia (72.4%) reported in the present study was inconsistent with previous studies. The difference in the timing of the study could have been the reason for this discrepancy with earlier studies reporting a much lower prevalence. Earlier, smartphone and internet penetration was reportedly low. Moreover, lack of validated questionnaire could as well have reported contrasting results but a rapid rise has been reported in recent studies with some studies among medical students reporting addiction as high as 92% [14]. Anxiety of separation, and loneliness at the start of medical school could as well contribute to smartphone addiction and nomophobia [16]. Similar findings have been reported from China [17] and Lebanon [18].

Our study reported pain in the wrist or on the back of neck due to excessive smart phone use among 39.2% participants. This may lead to future physiological and psychological complications. The result of the present study reported a higher percentage of male to female smartphone addicts similar to a study among Iranian and Chinese medical students but unlike others reporting higher female addiction [17, 19, 20]. The evolution of internet and smartphone based games and its gaining popularity among males could be a cause of such a finding [18, 21]. Our present study reported that females used smartphones mostly for communication and social networking. Males used smartphone mostly for communication and gaming concurrent with a study from China [17]. The similarity could have been a result of parallel nature of either gender of similar age and field of study. The present study is not in a situation to speculate the similarity and a need for further studies is recommended to resolve the inconsistent prevalence of smartphone addiction among gender. More internet and gaming addiction studies could prove such a finding in Nepal’s context.

Studies have shown a higher number of females accepting smartphone addiction than male participants similar to present study [19]. However, the present study reported a higher male mean addiction score than females (30.23 ± 9.40:28.89 ± 8.63).

Studies have shown bidirectional influence of smartphone and psychiatric conditions. Some suggest smartphone addiction can cause insomnia, restlessness, stress, depression and impulsive behavior, [22, 23] whereas some studies show these disorders could facilitate smartphone addiction [24]. These conflicting evidences warrant further studies. In the present study, we postulated impulsive overuse of smartphone as probable cause of smartphone addiction. Using smartphone for more than 5 h a day during weekdays and self-perception of being addicted to smartphone were found to be corelated with smartphone addiction. This finding was similar to the one reported in Lebanon [18]. Excessive use could be a sign of addiction and carefree nature. Almost 43% participants reported being complained about excessive use of smartphone by people nearby. The present study reported smartphone overuse among 16.8% (42/250) participants and its association with addiction similar to a study among medical students in Iran. The similarity could have been a result of homogenous sample population [25]. In the present study, the participants were instructed to self-report their duration of smartphone use where participants could have had difficulty recalling the past. This recall bias is one of the limitations of this study.

Self-acceptance was another factor most closely associated with smartphone addiction reinforcing the fact that self-admission and self-esteem had direct effect on mobile phone addiction. Self-acceptance was found to be the biggest predictor of smartphone addiction. This was consistent with study from Korea [23] unlike from China [26].

The study showed a significant association between gender and smartphone addiction (*p* < 0.01) unlike one reported in India as recent as 2016 [27]. The association could have been observed because of larger female sample population. More such studies are necessary to confirm such fact. Previous educational institute namely government or private academic institutions showed no correlation with smartphone addiction in either sexes.

Decreasing tendency of smartphone addiction with progressive year has been observed in India [28, 29]. Further studies involving all level of undergraduate students is recommended to establish such a fact.

Studies have shown the use of smartphone for learning purpose as a protective factor over unproductive use for smartphone addiction [30, 31]. In our study only 12/250 (M = 5 and F = 7) participants used smartphone solely for study purpose. This could as well be a reason for higher percentage of smartphone addiction in the current study. Use of smartphone for social networking was the commonest cause of smartphone addiction.

## Conclusion

Smartphone addiction was common among the investigated medical students. Excessive use of smartphone may lead to the risk of addiction. Responsible use can benefit individuals and any form of addiction can be checked by a strong will. We suggest a need for intervention to reduce smartphone addiction among undergraduate medical students probably incorporating health education about the use of mobile phone. Young people should be advised and educated about conscientious use of smartphone to avoid detrimental impact on daily life. Further studies will be required to reveal smartphone addiction in different levels of education.

## Data Availability

The datasets obtained and/or analyzed during the current study are not publicly available due to confidentiality consent of the study but can be obtained from the corresponding author on reasonable request.

## Abbreviation

SAS-SV: Smartphone addiction scale- short version
